# Loss-of-function mutations in *ASIP* and *MC1R* are associated with coat colour variation in marsupials

**DOI:** 10.1098/rsbl.2025.0302

**Published:** 2025-10-22

**Authors:** Ryan Sauermann, Bronwyn Fancourt, Tim Faulkner, Hayley Shute, Dean Reid, Andrew J. Pask, Charles Yakov Feigin

**Affiliations:** ^1^ School of BioSciences, The University of Melbourne, Melbourne, Victoria, Australia; ^2^ School of Environmental and Rural Science, University of New England, Armidale, New South Wales, Australia; ^3^ Department of the Environment, Tourism, Science & Innovation, Queensland Parks and Wildlife Service, Toowoomba, Queensland, Australia; ^4^ Australian Reptile Park & Aussie Ark, Somersby, New South Wales, Australia; ^5^ School of Agriculture, Biomedicine and Environment, La Trobe University, Melbourne, Victoria, Australia

**Keywords:** pigmentation, convergent evolution, genomics, loss-of-function, coat colour, melanism, xanthism, quoll, marsupial mole, Tasmanian devil

## Abstract

Pigmentation in mammalian hair follicles is governed in part by interactions between agouti signalling protein (ASIP) and the melanocortin 1 receptor (MC1R). The most common coat colours in mammals result from alternating bands of dark eumelanin and light phaeomelanin within individual hair shafts. However, coats dominated by a single melanin have arisen several times. Here, we examine the genetic basis of two instances in marsupials: a melanistic morph of the eastern quoll (*Dasyurus viverrinus*) found at high frequency in the wild, and a rare case of fixed xanthism in the marsupial moles. In eastern quolls, we identify a deletion encompassing the *ASIP* start codon which was found to be homozygous only in the melanistic animals examined. This mutation appears to be convergent with that recently discovered in its dark-coated relative, the Tasmanian devil (*Sarcophilus harrisii*). Conversely, we show that a non-sense mutation which severely truncates *MC1R* in the southern marsupial mole (*Notoryctes typhlops*) is a candidate driver of its pale-yellow coat. Together with other recent findings, our results suggest that loss-of-function mutations have occurred repeatedly within the marsupials, representing a mechanism underpinning coat colour variation.

## 1. Introduction

**Figure 1.**
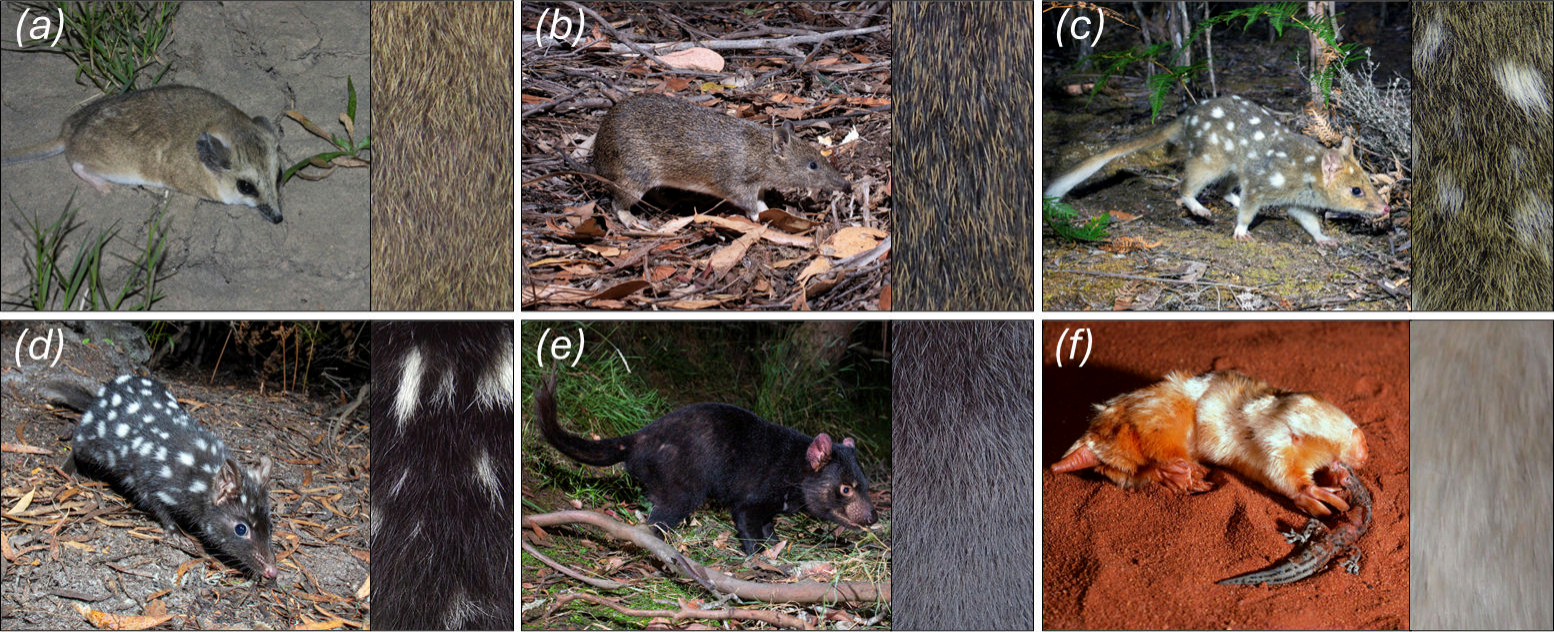
Illustration of marsupial coat colour variation. Species such as the (*a*) fat-tailed dunnart (*Sminthopsis crassicaudata*) and (*b*) southern brown bandicoot (*Isoodon obesulus*) exhibit the typical mammalian agouti pattern with alternating bands of phaeomelanin and eumelanin. (*c,d*) The eastern quoll (*Dasyurus viverrinus*) has two common colour morphs, (*c*) a wild-type ‘fawn’ morph with an agouti background coat and (*d*) a melanistic ‘black’ morph. (*e*) The Tasmanian devil (*Sarcophilus harrisii*) has a solid black background coat. (*f*) The marsupial moles (illustrated here by the southern species, *Notoryctes typhlops*) typically have a pale-yellow coat. Credits for animal photographs: (*a*) Lachlan Copeland, (*b–e*) Brett Vercoe and (*f*) Mike Gillam.

Coat colour in mammals results from the quantities, transport and spatial distributions of two types of pigment, dark eumelanin and light phaeomelanin [1]. Within the hair follicle, these pigments are regulated by the secretion of agouti signalling protein (ASIP) from dermal papilla (DP) cells, which binds to the melanocortin 1 receptor (MC1R) on the surface of pigment-producing melanocytes [2]. MC1R constitutively promotes the production of eumelanin, a function that is enhanced by its agonist, α-MSH. ASIP functions as an inverse agonist, both competing with α-MSH to bind MC1R and reducing MC1R’s activity, leading to a shift towards production of phaeomelanin [2]. The most common dorsal coat colours in mammals, ranging from grey to light brown, result from alternating bands of phaeomelanin and eumelanin within individual hair shafts, a phenotype called agouti (figure 1*a*–*c*) [1]. In contrast, patterns that span many follicles, including stripes and spots, are regulated by mechanisms that establish spatial information across whole skin regions, such as reaction–diffusion systems and long-range morphogens [3].

Loss of a single type of melanin has arisen frequently in mammals through mutations that modify or inhibit regulators of pigment production. Among the most common examples in mammals is melanism, a phenotype in which eumelanin is produced exclusively or in elevated quantities, leading to dark brown or black hair. Genetic studies have linked many cases of melanism, with either loss-of-function (LoF) mutations in *ASIP* or gain-of-function (GoF) mutations in *MC1R*, including field and pocket mice, multiple felid and squirrel species and domestic sheep [4–8]. Coat colours driven predominantly by phaeomelanin appear to be rarer. Examples include pale cream coloration in Australian cattle dogs associated with a mutation in *MC1R*’s promoter, as well as the yellow labrador, golden retriever and Irish setter, whose alleles exhibit a truncation at the MC1R C-terminus [9–12]. Such phenotypes can be classified as xanthism or xanthochromism when phaeomelanin is yellow in colour, or erythrism when it has a more orange to red hue [13].

Coats dominated by one type of melanin have arisen multiple times in marsupials, including instances where they have become fixed (figure 1*d*–*f*). For example, we recently showed that in the Tasmanian devil (*Sarcophilus harrisii*), a LoF mutation in *ASIP* likely contributes to the species’ distinctive, black coat (figure 1*e*) [14]. A similar phenotype exists as a high-frequency polymorphism in the closely related and sympatric eastern quoll (*Dasyurus viverrinus*; figure 1*c*,*d*) [15]. The marsupial moles (genus *Notoryctes*), which comprise two cryptic, fossorial species native to Australia’s great deserts, exhibit a cream to pale-yellow colour (figure 1*f*) [16]. This phenotype represents a rare case of fixed xanthic coloration in a wild mammal.

Here, we examine the genomic basis of coat colour in the eastern quoll and southern marsupial mole. First, we identify a deletion at the *ASIP* locus in eastern quolls that may underpin their melanistic morph. This mutation appears convergent with that found in the related Tasmanian devil [14]. Furthermore, by examining the *MC1R* locus across agreodont marsupials (comprising the orders Dasyuromorphia, Peramelemorphia and Notoryctemorphia) [17,18], we identify a non-sense mutation that severely truncates the receptor as a candidate for the marsupial mole’s distinctive pale-yellow coat. Together with recent findings in other marsupials, our findings illustrating that LoF changes in core pigment-regulating genes are potentially important mechanisms for the evolution of coat colour in this lineage.

## 2. Results

### (a) Partial *ASIP* deletion as a candidate driver of eastern quoll melanism

We recently generated a reference genome for the eastern quoll (DasViv_v1.0) [14]. This animal exhibited the wild-type ‘fawn’ colour morph, which has agouti-banded fur, punctuated by white spots (figure 1*c*). A melanistic morph, which exhibits black background fur while retaining white spots (figure 1*d*), is also found at high frequency in both wild and captive populations [19–21]. Given that *ASIP* LoF is a driver of melanism in other mammals and is expected to be recessive based on its molecular function, we first asked whether our fawn-coloured reference individual may have been a carrier for such a mutation. To this end, we generated a new, haplotype-phased genome of the eastern quoll by reassembling PacBio HiFi and Omni-C reads that were used to produce the previous pseudohaplotype genome. The two resulting haplotypes were named DasViv_v2.0_hap1 and DasViv_v2.0_hap2 (hereafter, referred to as haplotype 1 and haplotype 2, respectively). Scaffold metrics for each haplotype were comparable to those of DasViv1.0 (electronic supplementary material, table S1).

**Figure 2.**
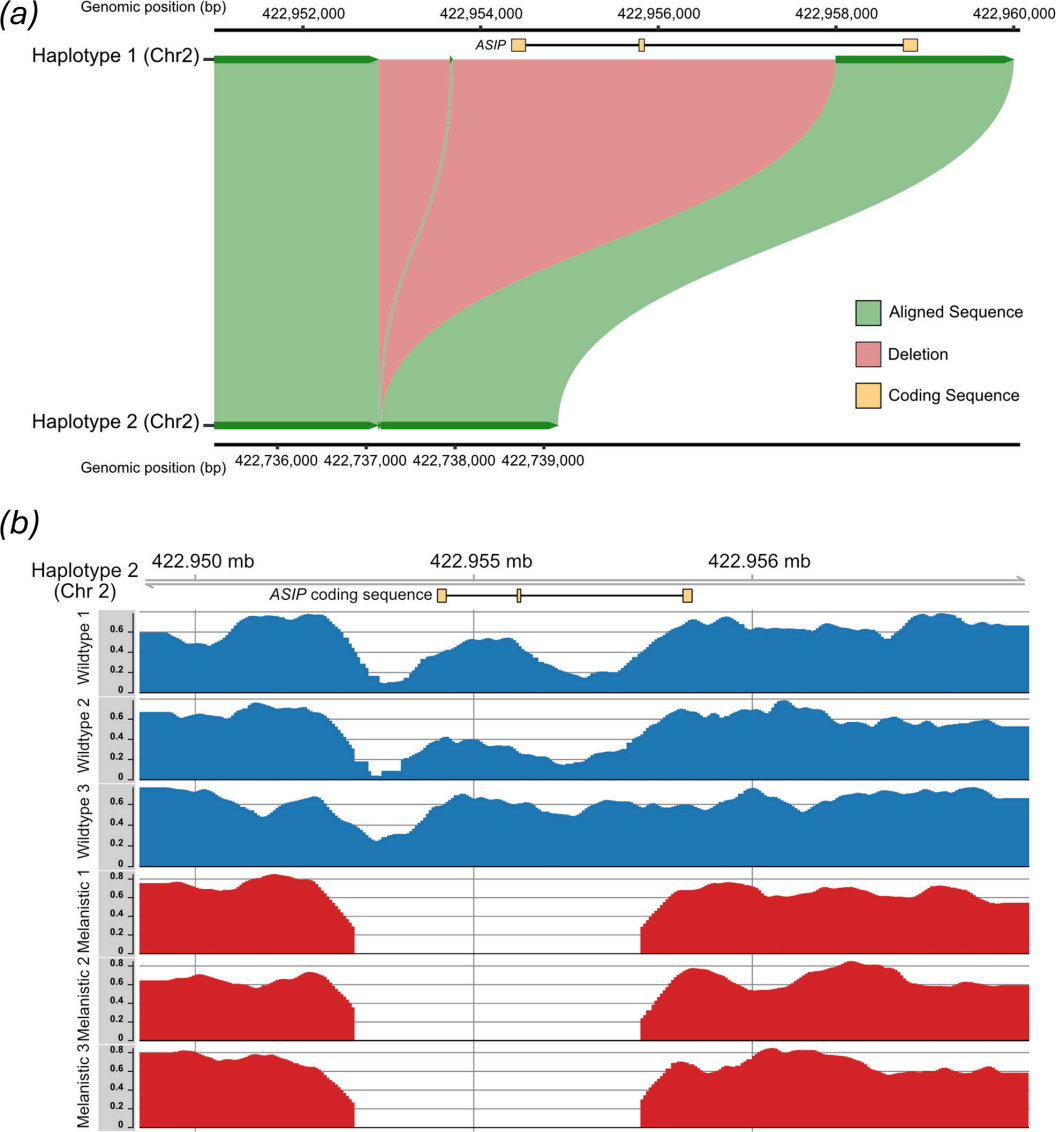
(*a*) Chromosomal alignment of phased eastern quoll haplotype assemblies at the *ASIP* locus, centred on the deletion region. Green ribbons represent sequences aligned between haplotype 1 (top) and haplotype 2 (bottom). Red ribbons represent the putative deletion region, which is present in haplotype 1 but absent from haplotype 2. Coding exons (i.e. CDS annotations) for *ASIP* are shown in yellow above the alignment, illustrating that the first two coding exons are deleted. (*b*) Mapping coverage of wild-type and melanistic eastern quolls across the *ASIP* locus. Melanistic quolls (red) show no read coverage across the 4.7 kb deletion region, indicating homozygosity for this deletion, in contrast to wild-type animals (blue).

To facilitate examination of the *ASIP* locus, we next annotated this gene in each eastern quoll haplotype assembly, as well as assemblies of several related marsupials (electronic supplementary material, table S2). While the *ASIP* allele annotated on haplotype 1 contained a complete coding sequence as in other marsupials with typical agouti banded hair, annotation of the first two coding exons on haplotype 2, including the start codon, was unsuccessful (electronic supplementary material, data S1). Blastn searches for these exons similarly returned no hits against haplotype 2 [22]. Given this, we next extracted the genomic region surrounding the expected locations of these exons from both eastern quoll haplotypes. Alignment of these sequences revealed an approximately 4.7 kb stretch of sequence missing in eastern quoll haplotype 2 and encompassing the first two coding exons (figure 2*a*, electronic supplementary material, data S2). This observation indicated that the individual used to generate the eastern quoll reference genome was indeed heterozygous for a presumptive LoF mutation at the *ASIP* locus. Interestingly, this eastern quoll *ASIP* deletion appears to be convergent with the fixed *ASIP* deletion recently identified in a closely related dasyurid marsupial, the Tasmanian devil. Both deletions share a similar upstream break point, though the devil mutation only encompasses the first coding exon (electronic supplementary material, data S2) [14].

To further explore this finding, we sequenced genomic DNA from fawn (*n* = 3) and black morph (*n* = 3) eastern quolls and visualized mapping coverage against eastern quoll haplotype 1 (which retains the intact *ASIP* locus). This revealed that the examined black morph quolls showed zero mapping coverage over the genomic interval that corresponded to the *ASIP* deletion on eastern quoll haplotype 2, while the examined fawn morph quolls showed high coverage (figure 2*b*). These observations may be consistent with recessive melanism driven by *ASIP* LoF in eastern quolls. However, additional sampling across the eastern quoll population is needed to confirm this mode of inheritance.

### (b) Truncation of *MC1R* may contribute to pale-yellow coat colour in marsupial moles

Another instance among agreodonts where agouti banding has been lost is in the marsupial moles. Comprising two species, the northern and southern marsupial moles (*Notoryctes caurinus* and *Notoryctes typhlops*, respectively), this desert-dwelling group has evolved a suite of convergent adaptations with eutherian moles to facilitate its fossorial lifestyle, including highly reduced eyes and modified forelimbs, which they use to ‘swim’ through loose sands [17,23]. They are among the most rarely observed mammals in Australia, with only a handful of reported sightings each decade. All documented individuals have been found to exhibit a cream to pale-yellow coat, with no obvious sign of dark eumelanin deposition (figure 1*f*) [24]. We recently generated a reference genome for the southern marsupial mole, and thus sought to explore the basis of this species’ unique coat colour [17]. Given the constitutive function of MC1R in eumelanin production, we hypothesized that a LoF at this locus might contribute to its apparent absence in marsupial mole hair.

**Figure 3.**
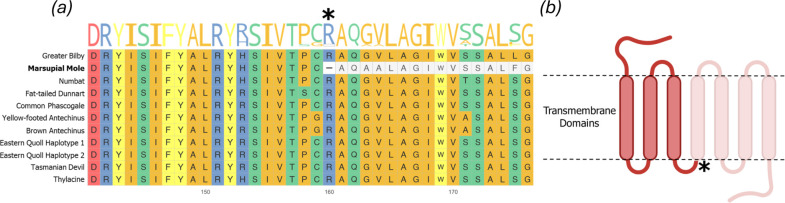
(*a*) Partial view of aligned amino acid sequences of MC1R orthologues across examined marsupials. Colours indicate residues with similar side chain properties. The sequence logo represents the frequency of different residues at each position. The asterisk indicates the location of the identified non-sense mutation in the marsupial mole, with the truncated bases shown in grey. (*b*) A diagram of the MC1R protein showing domains ablated by this change.

As *MC1R* was successfully annotated in all marsupial genomes tested, we translated and aligned the amino acid sequences of its orthologues. While *MC1R* was overall highly conserved across these species, a single gap was present, exclusively in the marsupial mole. Examination of the nucleotide alignment at this position showed that the gap was due to an in-frame stop codon at position 160, in place of a highly conserved arginine residue (R160X; figure 3*a* and electronic supplementary material, data S4). Based on the UniProt structural annotation of mouse MC1R (A0A0B6VTJ4), truncation from the homologous residue (R158 in mouse) is expected to ablate approximately 50% of the protein’s length, including four of seven transmembrane (TM) domains (figure 3*b*) [25,26].

## 3. Discussion

Here, we provide evidence that repeated LoF mutations in core pigment-regulating genes have contributed to coat colour variation within and between marsupial species. In eastern quolls, we identified a putative *ASIP* LoF allele, which was found to be homozygous only in examined melanistic animals. Ablation of the only annotated start codon and two complete coding exons likely prevents translation of a functional protein product, resulting in a complete loss of ASIP’s function as an inverse agonist of MC1R. *ASIP* LoF mutations have previously been associated with melanism in various other mammals [4–6]. Intriguingly, this mutation appears to be convergent with a similar deletion recently identified in its close relative, the Tasmanian devil (electronic supplementary materials, data S1 and S2) [14,27]. While differing in size (the devil deletion encompasses only the first coding exon), we expect the effects of these mutations to be similar, due to the loss of the start codon. Of note, the 5′ breakpoints of the Tasmanian devil and eastern quoll deletions are very close in position (electronic supplementary material, data S12). Sequence similarity between genomic regions flanking the deletion region was markedly higher between the two eastern quoll haplotypes than with the Tasmanian devil orthologous region, including nine indels all of which were species specific. Additionally, maximum likelihood phylogenies generated from the approximately 1.5 kb sequences immediately flanking each side of the eastern quoll *ASIP* deletion and orthologous regions in dasyurids placed both eastern quoll haplotypes together, to the exclusion of the Tasmanian devil (electronic supplementary materials, figure S1 and data S3). Together, these findings suggest that the eastern quoll *ASIP* deletion arose independently from that in the Tasmanian devil and far more recently than the divergence between these two sister genera. Given this, we propose that the Tasmanian devil and eastern quoll *ASIP* deletions likely represent convergence, rather than a shared ancestral variant.

Dark coat has evolved in other marsupials, including brushtail possums (*Trichosurus vulpecula*), a phenotype recently shown to be driven by a mutation in the *ASIP* coding sequence [28]. The co-occurrence of multiple dark-coated species in Tasmania was noted by Bond *et al.* [28], with several possible explanations explored [28]. One hypothesis proposed was a fitness benefit for darker fur colour in Tasmania’s humid climates, in line with Gloger’s rule [29]. Tasmania became separated from mainland Australia around 14 000 years ago at the end of the last glacial maximum, and represents the southern end of the species’ historical range [30]. However, black morph eastern quolls were readily observed across southeastern Australia prior to their extirpation from the mainland in the late twentieth century [21,31]. Black morph eastern quolls have thus existed in a relatively wide range of climates. Given this, it is unclear whether humidity can explain the persistence of melanistic eastern quolls. Melanism as a cryptic morph has also been suggested as an historical adaptation to avoid predation by the recently extinct Tasmanian tiger (*Thylacinus cynocephalus*) [28,32]. Indeed, the melanistic Tasmanian devil also shared its historical range with the thylacine. However, agouti-banding is nearly universal among mammals, including in nocturnal species. Therefore, it is dubious whether melanism provides effective camouflage for these species. Population-level studies will likely be needed to better understand the fitness implications of *ASIP* LoF and melanism in marsupials.

In stark contrast to the eastern quoll and Tasmanian devil, marsupial moles appear to have lost eumelanin production in their fur altogether. Our analyses indicate that an early stop codon that truncates ASIP’s receptor, MC1R, may be a contributing factor. Truncation of the rabbit orthologue of *MC1R* with CRISPR-Cas9 results in a light yellow coat, due to the loss of eumelanin deposition into the hair shaft [33]. These mutations ablated at least half of the TM domains essential for MC1R function. A similar, though less severe mutation has been identified in dog breeds with yellow coats, such as yellow labrador and golden retrievers [11]. This suggests that the similar natural truncation of marsupial mole *MC1R*, which deletes four out of seven TM domains, may contribute to its distinctive pale-yellow coat colour. Notably, rabbits with *MC1R* truncations exhibit a more striking yellow hue, reminiscent of spontaneous xanthism/xanthochromism observed in various wild mammals [9,10,13,33], while the dorsal fur of marsupial moles is a paler yellow or cream colour (figure 1*f*). Marsupial moles may also therefore exhibit a degree of hypopigmentation. However, regions of the marsupial mole’s fur, such as the posterior aspect of the hindlimbs, show more prominent yellow coloration (figure 1*f* and electronic supplementary material, figure S2). The genus *Notoryctes* comprises two distinct species, the southern marsupial mole (examined here) and northern marsupial mole, both of which share a similar coat colour [24]. Molecular studies indicate that these sister species diverged around 4.6 million years ago [23], strongly suggesting that the loss of eumelanin production through *MC1R* LoF may have arisen prior to their divergence. While these animals are fossorial, their lighter coloration may provide some camouflage (depending on the colour of local sands) or may contribute to thermoregulation when the animal briefly emerges on the surface [34]. Alternatively, this may reflect a relaxation of constraint on pigmentation pathways in the context of a subterranean lifestyle.

Our results suggest that LoF mutations in the pigment regulators *ASIP* and *MC1R* contribute to coat colour in the eastern quoll and marsupial mole, respectively. Taken together with recent findings in related marsupials, they suggest that LoF mutations in core pigment-regulation genes may represent an important mechanism for the evolution of coat colour in this lineage. Our work further invites new investigations into the potential adaptive implications of such mutations.

## 4. Methods

### (a) Genome assembly

PacBio HiFi (approx. 97.68 Gb) and Omni-C (approx. 126 Gb) reads (NCBI BioProject PRJNA75870) previously used to produce a pseudohaplotype assembly of a female, fawn morph eastern quoll were reanalysed to produce a new haplotype-phased assembly [14,27]. Briefly, PacBio reads were provided as input to hifiasm v. 0.15.4-r343 with default parameters to produce primary scaffolds [35]. Next, Omni-C reads were aligned against the assembly with bwa mem v. 0.7.17. The alignment and assembly were then provided to the HiRise pipeline (Dovetail Genomics; Scotts Valley, CA, USA) yielding two haplotype assemblies [36]. Assembly quality for each haplotype was compared to that of the original pseudohaplotype genome using BUSCO v. 5.8.2 (parameters -m genome -l mammalia_odb10) and the stats.sh script provided by the bbmap package with default parameters (electronic supplementary material, table S1) [37,38]. Scaffold metrics and BUSCO gene recovery were roughly the same between the original pseudohaplotype assembly (DasViv_v1.0) and each haplotype assembly (DasViv_v2.0_hap1 and DasViv_v2.0_hap2, respectively) with marginal improvements in ‘Complete Single Copy’ and ‘Missing’ orthologue categories when the complete haplotype-phased assembly is taken together.

### (b) Identification and alignment of *ASIP* and *MC1R* orthologs

To identify orthologues of *ASIP* and *MC1R* across agreodont marsupials (electronic supplementary material, table S2) [14,27,39–45], we used the programme LiftOff v. 1.6.3 [46] (default parameters) to perform lift-over annotations. To avoid potential biases introduced by using a reference species with divergent pigmentation, we used the high-quality RefSeq gene models from the agouti-patterned yellow-footed antechinus (*Antechinus flavipes*) [39]. CDS sequences of *ASIP* and *MC1R* were then extracted from each genome using Gffread v. 0.9.12 [47] and aligned using the MAFFT web server (https://mafft.cbrc.jp/alignment/software/) [48]. Searches for *ASIP* coding regions missing in eastern quoll haplotype 2 were performed using Blastn v. 2.16.0+ [22]. As no hits were returned, no results are shown. MC1R orthologues from all examined marsupial genomes were translated and realigned with MAFFT. Visualization of the MC1R amino acid alignment was performed using the R package ggmsa v. 1.13.1 [49].

### (c) Alignment and visualization of the *ASIP* genomic locus

Using the location of annotated *ASIP* exons identified by LiftOff, we extracted the *ASIP* locus from both eastern quoll haplotypes as well as the Tasmanian devil, which were aligned using the MAFFT web server as above. To produce the visualization shown in figure 2*a*, the *ASIP* locus in eastern quoll haplotypes 1 and 2 were realigned with minimap2 v. 2.28-r1209 (parameters: -x asm20 --eqx --secondary = no c) and plotted using the R package SvbyEye v. 0.99.0 [50,51].

### (d) Phylogenetics of *ASIP* flanking sequences

To help distinguish convergent evolution of *ASIP* LoF from a shared ancestral deletion, we constructed maximum likelihood phylogenies of the sequences immediately flanking the deletion in the eastern quoll and Tasmanian devil, along with orthologous sequences from closely related dasyurid marsupials with highly contiguous assemblies of this region (electronic supplementary material, data S1). Briefly, using the annotated coordinates of the deletion region (see Identification and alignment of *ASIP* and *MC1R* orthologues), we extracted 1.5 kb of sequence upstream and downstream of the region in the eastern quoll, as well as the homologous sequences in the wild-type allele and genome assemblies of the Tasmanian devil, brown and yellow-footed antechinuses and fat-tailed dunnart using samtools faidx (electronic supplementary materials, table S2 and data S3). Upstream and downstream sequences were aligned using MAFFT v. 7.490 (--maxiterate 1000) [48]. Phylogenies were inferred using RAxML 8.2.12 with 1000 bootstrap replicates (-f a -p 2025 x 2025 -#1000 m GTRGAMMA), specifying the fat-tailed dunnart as the outgroup [52]. Trees were visualized using FigTree v. 1.4.4 (http://tree.bio.ed.ac.uk/software/figtree/).

### (e) Eastern quoll resequencing

Tissue samples of six eastern quolls of known colour morph (*n* = 3 fawn, *n* = 3 black; electronic supplementary material, table S3) were acquired as secondary use from the Aussie Ark captive breeding sanctuary (Somersby, NSW, Australia) and through prior field collections on Bruny Island under University of Tasmania Animal Ethics Committee Ethics Approval Permit A11655 and Department of Primary Industries, Parks, Water and Environment permits FA10042 and FA11050. DNA was extracted with the DNeasy Blood & Tissue Kit (Qiagen, cat. no. 69504). Libraries were prepared using the VAHTS Universal Pro DNA Library Prep Kit for Illumina (Vazyme, cat. no. ND610) and were sequenced to a depth of at least 25× coverage on an Illumina NovaSeq S4 flow cell by Azenta Life Sciences (Burlington, MA, USA).

Raw reads were filtered and trimmed using fastp (default parameters) and mapped against DasViv_v2.0_hap1, which retains a complete copy of the *ASIP* locus, using bwa-mem2 v. 2.2.1 (parameter -M) [53]. Alignments were filtered using samtools view v. 1.13 to retain only primary alignments with a MAPQ greater than 30 and properly paired reads (parameters -f 3 F 3340 -q 30) [54]. Normalization for mapping coverage and conversion to bedgraph format were performed using deepTools bamCoverage v. 3.5.5 (--scaleFactor 10 --normalizeUsing BPM --exactScaling --normalizeUsing BPM) [55]. Visualization of read coverage over the region shown in figure 2*b* was performed using the R package Gviz v. 1.50.0 [56].

## Data Availability

Eastern quoll haplotype assemblies 1 and 2 are available on NCBI in fasta format under BioProjects PRJNA1209419 and PRJNA1209418, respectively. Whole genome resequencing fastq reads are available under BioProject PRJNA1209406. All original code used in this study is attached as electronic supplementary materials in codes S1–S3. The marsupial mole photograph used in figure 1*f* is copyrighted (Mike Gillam/AUSCAPE, all rights reserved) and included here on a single-use, non-exclusive licence. Supplementary material is available online [57].
